# Light, Not Age, Underlies the Maladaptation of Maize and Miscanthus Photosynthesis to Self-Shading

**DOI:** 10.3389/fpls.2020.00783

**Published:** 2020-06-24

**Authors:** Robert F. Collison, Emma C. Raven, Charles P. Pignon, Stephen P. Long

**Affiliations:** ^1^Department of Plant Sciences, University of Oxford, Oxford, United Kingdom; ^2^Carl R. Woese Institute for Genomic Biology, University of Illinois, Urbana, IL, United States; ^3^Department of Crop Sciences, University of Illinois, Urbana, IL, United States; ^4^Department of Plant Biology, University of Illinois, Urbana, IL, United States; ^5^Lancaster Environment Centre, Lancaster University, Lancaster, United Kingdom

**Keywords:** C_4_ photosynthesis, canopy, bioenergy, food security, quantum yield, shade acclimation, photosynthetic light-use efficiency, leaf aging

## Abstract

*Zea mays* and *Miscanthus × giganteus* use NADP-ME subtype C_4_ photosynthesis and are important food and biomass crops, respectively. Both crops are grown in dense stands where shaded leaves can contribute a significant proportion of overall canopy productivity. This is because shaded leaves, despite intercepting little light, typically process light energy very efficiently for photosynthesis, when compared to light-saturated leaves at the top of the canopy. However, an apparently maladaptive loss in photosynthetic light-use efficiency as leaves become shaded has been shown to reduce productivity in these two species. It is unclear whether this is due to leaf aging or progressive shading from leaves forming above. This was resolved here by analysing photosynthesis in leaves of the same chronological age in the centre and exposed southern edge of field plots of these crops. Photosynthetic light-response curves were used to assess maximum quantum yield of photosynthesis; the key measure of photosynthetic capacity of a leaf in shade. Compared to the upper canopy, maximum quantum yield of photosynthesis of lower canopy leaves was significantly reduced in the plot centre; but increased slightly at the plot edge. This indicates loss of efficiency of shaded leaves is due not to aging, but to the altered light environment of the lower canopy, i.e., reduced light intensity and/or altered spectral composition. This work expands knowledge of the cause of this maladaptive shade response, which limits productivity of some of the world’s most important crops.

## Introduction

C_4_ grasses of the Andropogoneae represent some of the most important cultivated plants on the planet, making up a significant proportion of our food and fibre production, as well as providing major bioenergy crops. All members of this monophyletic tribe use the NADP-ME subtype of C_4_ photosynthesis, with some species using substantial PCK activity. This tribe includes crops such as *Saccharum officinarum* L. (sugarcane), the greatest producer of harvested biomass globally, and *Zea mays* L. (maize), the single largest source of grain globally (Christin et al., 2009; Welker et al., 2014; FAOSTAT, 2017). Other C_4_ NADP-ME crops of this tribe are highly productive in the face of extreme climatic conditions, and thus vital to food production in drought prone environments. *Sorghum bicolor* (Lu.) Moench (sorghum), for instance, is the second most extensively cultivated crop plant in Africa behind *Z. mays* thanks to its high drought tolerance (FAOSTAT, 2017; Hadebe et al., 2017). The tribe also includes the most productive temperate biomass crop known, *Miscanthus × giganteus* Greef et Deu. (Heaton et al., 2008; LeBauer et al., 2018).

The theoretical maximum efficiency of conversion of solar energy to biomass is 6% for C_4_ compared to 4.6% for C_3_ photosynthesis at 30°C and 380 ppm atmospheric CO_2_: this improved photosynthetic light-use efficiency contributes to higher yields in C_4_ crops (Zhu et al., 2008). The key metric for photosynthetic light-use efficiency is the quantum yield of CO_2_ assimilation, i.e., the mol CO_2_ assimilated per mol photons of light. In a typical light-response curve, the quantum yield of CO_2_ assimilation is greatest when light is limiting, and declines at high light as photosynthesis becomes light-saturated. The maximum quantum yield of CO_2_ assimilation (*ϕCO_2 max, app_*), achieved under limiting light, is therefore paramount for the productivity of shade leaves. Shade leaves are estimated to contribute around 50% of total canopy carbon gain in field crops and may represent >80% of leaves in a dense crop stand (Baker et al., 1988; Long, 1993; Hikosaka et al., 2016). Accordingly, leaves of most plants respond to increasing shade by maintaining or increasing *ϕCO_2 max, app_* so that they can make maximum use of the limited light. However, in *Z. mays* and *M. × giganteus* a significant decrease in *ϕCO_2 max, app_* has been observed in leaves as they become progressively shaded by new leaves forming above them, with a projected cost of up to 10% of potential canopy CO_2_ assimilation (Pignon et al., 2017). With the continued trend of increasing planting density this loss will likely increase into the future (Lobell et al., 2014).

Shade acclimation in C_4_ species has been studied primarily by comparing plants grown in high vs. low light (Tazoe et al., 2008; Sales et al., 2018; Sonawane et al., 2018). On this basis, it has been observed that C_4_ species have relatively poor acclimation to shade relative to C_3_ species (Sage and McKown, 2006), but C_4_ grasses which use the NADP-ME subtype, such as *Z. mays*, acclimate to shade more readily than those using NAD-ME or PEP-CK subtypes (Sonawane et al., 2018). However, in these studies the shaded leaves grow while the entire plant is shaded, such that their entire development occurs in the shade. In crop fields, leaves form in full sunlight, but then become progressively shaded after they have completed development as new leaves form above them (Yabiku and Ueno, 2019). Less is known about acclimation in this situation, which is particularly relevant to crop productivity. Plasticity to shade in this context is more limited, since leaves are already fully formed and acclimated to high light before becoming shaded. In grasses, plasticity of key physiological traits, such as leaf nitrogen (N) content, declines with increasing leaf age (Niinemets, 2016a). In addition, shade in the lower canopy is not simply reduced light quantity, but also altered spectral light composition, with relative depletion of red and blue, and enrichment of green and near infrared, plus an increased incidence of light fluctuations due to sun flecks (Pearcy, 1990). Leaves of NADP-ME C_4_ grasses lose photosynthetic efficiency under these conditions (Kromdijk et al., 2008; Kubasek et al., 2013; Pignon et al., 2017).

The two major distinctions between a sun and shade leaf in a C_4_ grass canopy are leaf age and light environment. Understanding whether the decline of photosynthetic efficiency in shade leaves results from age, light environment, or both, is an important first step in devising strategies to overcome this costly maladaptation in these key crops. For instance, efforts to optimize canopy architecture have involved producing crops with more erect (Perez et al., 2018; San et al., 2018) or more transparent (Slattery et al., 2016; Walker et al., 2018) leaves that increase light availability at the bottom of the canopy to increase canopy photosynthesis (Zhu et al., 2010). This strategy may not be as effective in C_4_ grass canopies if the leaves at the bottom of the canopy have lost efficiency in low light due to age, and so have limited ability to utilize the increased levels of *PPFD* enabled by these canopy alterations.

Classically, leaf shade adaptation involves maintaining maximum quantum yields on an absorbed light basis (*ϕCO_2 max, abs_*), and increasing leaf light absorbance (α) through increased chlorophyll concentration, to deliver increased photosynthesis in the shade. However, prior evidence has shown the reverse to occur in *Z. mays* and *M. x giganteus*, with a decrease in *ϕCO_2 max, abs_* and significant cost to canopy photosynthesis (Pignon et al., 2017). Here, we tested the following hypothesis: chronological age is responsible for the loss of maximum quantum yields of photosynthesis in field plots of the C_4_ NADP-ME grasses *Z. mays* and *M. x giganteus*. Leaves were collected from the top and bottom of the canopy at the south exposed edge and at the centre of field plots of these crops, such that lower canopy leaves from both plot positions were of the same chronological age, but only those at the plot centre were shaded. This enabled separation of the effects of environment and chronological age on differences in photosynthetic efficiency between sun and shade leaves in a field production setting. The maximum quantum yield of CO_2_ assimilation, and its underlying physiological drivers, were determined from leaf gas exchange, modulated chlorophyll fluorescence and light absorbance measurements.

## Materials and Methods

### Plant Material

Measurements were taken on *Zea mays* and *Miscanthus* × *giganteus*. Leaves were collected from >1 ha plots of a high-yielding modern *Z. mays* hybrid as described previously (Pignon et al., 2017) on the University of Illinois South Farms (40°02′N, 88°14′W, 216 m above sea level), and leaves of *M.* × *giganteus* (“Illinois” clone) from 4 ha plots on University of Illinois Energy Farm (40°07′N, 5 88°21′W, 228 m above sea level) as described previously (Joo et al., 2017). Soils at these sites are deep Drummer/Flanagan series (a fine silty, mixed, mesic Typic Endoaquoll) with high organic matter typical of the central Illinois region of the Corn Belt (Smith et al., 2013). Both plots were rainfed. The *M.* × *giganteus* plots were 9 years old, with a stem density of about 100 tillers m^–2^; these plots were unfertilized. *Z. mays* was sown in early May at a density of 75,000 seeds ha^–1^. Prior to planting, 140 kg [N] ha^–1^ was applied, in line with regional production practice.

Measurements were taken between July 26 and August 06 of 2018. Leaves were cut pre-dawn at the base, then the base was submerged in water and re-cut to prevent air blockage in the xylem as described in Pignon et al. (2017). Removing leaves from plants in this way has been shown not to bias photosynthetic measurements (Leakey et al., 2006). Leaves were then brought back to the laboratory, where they remained in low light until measurement. This procedure avoided any photoinhibition or transient water stress that could develop differentially in shade and sun leaves over a day.

Leaves were sampled from two canopy positions (upper and lower) and two plot positions, centre and the south edge. For each plant sampled, two leaves were collected; an upper canopy leaf, defined as the youngest fully expanded leaf, indicated by a fully emerged ligule, and a lower canopy leaf; the seventh counting down from the first fully emerged leaf. This ensured that within a species and canopy position, leaves from the plot centre and edge were of the same age. The lower canopy leaves in the plot centre were strongly shaded, whereas lower canopy leaves at the plot’s edge were not. The south edge of the plot was chosen since on clear sky days these leaves were exposed to sunlight for 12 h per day.

### Measurement of Photosynthesis

Portable photosynthetic gas exchange systems (LI 6400 and LI 6400-40 modulated fluorescence chamber head; LI-COR, Inc., Lincoln, NE, United States) were used to measure CO_2_ and water vapor exchange on a 2 cm^2^ area of each leaf, along with modulated chlorophyll fluorescence, in the system’s controlled environment leaf cuvette. Air temperature was controlled at a constant 25.0°C, chamber [CO_2_] at 400 ppm, and water vapour pressure deficit at 1.6–2.4 kPa.

The measurement sequence began with estimation of maximum dark-adapted quantum yield of PSII photochemistry (*F_*v*_/F_*m*_*). A photosynthetic light response curve was generated as follows: integrated LEDs emitted uniform light consisting of 10% blue (465 nm wavelength) and 90% red (635 nm wavelength) across the leaf surface. In order to limit photoinhibition caused by sudden exposure to saturating light on enclosure in the cuvette, leaves were first subjected to a photosynthetic photon flux density (*PPFD*) of 100 μmol m^–2^ s^–1^ for 5 min, and subsequently exposed to 2000 μmol m^–2^ s^–1^ for 30–60 min until *A* reached a steady-state. *PPFD* was then decreased from 2000 in steps to 1500, 1000, 500, 200, 180, 160, 140, 120, 100, 80, 60, 40, 20, and 0 μmol m^–2^ s^–1^. Each *PPFD* step lasted 5–10 min to allow *A* to reach a steady state before measuring. Steady-state gas-exchange was recorded at each level of *PPFD* and used to calculate *A* (von Caemmerer and Farquhar, 1981). Modulated fluorescence measurements were made at each level of *PPFD* to determine the operating quantum yield of PSII (*ϕ_*PSII*_*) using a multiphase flash protocol (Loriaux et al., 2013). In turn, *ϕ_*PSII*_* was used to calculate the rate of linear electron flux through *PSII* (*J*), using measured values for leaf fractional absorptance of photosynthetically active photon flux (α, described below) and assuming a photon partitioning factor of 0.4 for PSII vs. PSI, i.e., accounting for increased photon partitioning to PSI to produce ATP through cyclic electron flux (Yin and Struik, 2012; Ver Sagun et al., 2019). Each *A*-*PPFD* response curve was fit to a four-parameter non-rectangular hyperbola using PROC NLIN (SAS v9.4, SAS Institute, Cary, NC, United States), which produced an asymptote, taken to represent light-saturated *A* (*A*_*sat*_), and a Y-intercept, taken to represent dark respiration (*R*_*d*_). The third parameter described light-limited *A* and the fourth parameter described the inflexion between light-limited and light-saturated *A* with increasing *PPFD.*

After gas-exchange measurements were completed, absorptance (α) was measured using an integrating sphere and associated spectrometer (Jaz-Spectroclip-TR, Ocean Optics, Largo, FL, United States) and operating software (Spectrasuite, Ocean Optics). α was weighted for 10% blue (465 nm wavelength) and 90% red (635 nm wavelength) incident light to match illumination in the gas-exchange chamber.

The maximum quantum yield of CO_2_ assimilation on an incident light basis (*ϕCO_2 max, app_*) was calculated from the slope of the linear regression of *A* against *PPFD* from 40 to 140 μmol m^–2^ s^–1^ using PROC GLM (SAS v9.4) (Yin et al., 2014; Pignon et al., 2017). This interval was chosen to account for the Kok effect where respiration increases at very low light levels (*PPFD* < 40 μmol m^–2^ s^–1^), and to avoid high light levels (*PPFD* > 140 μmol m^–2^ s^–1^) where *A* is no longer strictly light-limited causing deviations from the linear relationship of *A* and *PPFD.* The maximum quantum yield of CO_2_ assimilation on an absorbed light basis (*ϕCO_2 max, abs_*) was given by *ϕCO_2 max, app_* /α. Finally, the maximum quantum yield of CO_2_ assimilation on an absorbed light basis and corrected for concurrent changes in *ϕPSII* (*ϕCO_2 max, abs *PSII*_*) was calculated as in Yin et al. (2014). To test for alternative electron sinks to photosynthetic carbon metabolism, the slope of *A* vs. *J* was calculated for *PPFD* between 40 and 140 μmol m^–2^ s^–1^ using linear regression (SAS v9.4). The slope of this relationship gives the mol CO_2_ assimilated per mol electrons in linear electron flux (1/*k*) (Baker, 2008). Here *k* is the mol electrons through linear electron flux required for photosynthesis to fix one mol CO_2_. 1/*k* is an indicator of alternative energy sinks, where any reduction in 1/*k* is assumed to result from alternative energy sinks, including utilization of ATP and NADPH in processes other than photosynthetic carbon metabolism.

### Statistical Analysis

Data were analysed by ANOVA using PROC GLM (SAS v9.4), testing for the fixed effect of species (S effect: *Z. mays* vs. *M.* x *giganteus*), the fixed effect of canopy position (C effect: upper vs. lower canopy), and the fixed effect of plot position (P effect: centre vs. edge), along with all two-way interactions (S x P, S x C, P x C). This model was used to test for significant (*p* = 0.05 threshold) and marginally significant (*p* = 0.1 threshold) differences in the following traits: *ϕCO_2 max, app_*, *ϕCO_2 max, abs_*, *ϕCO_2 max, abs *PSII*_*, 1/*k*, *α*, *A*_*sat*_, *R*_*d*_, and *F_*v*_/F_*m*_*. Homogeneity of group variances was tested by Levene’s at *p* = 0.05 threshold in PROC GLM (SAS v9.4). Normality of Studentized residual distribution was tested by Shapiro–Wilk at *p* = 0.01 threshold in PROC UNIVARIATE (SAS v9.4). Replication was *n* = 8–16 in different traits.

## Results

**FIGURE 1:**
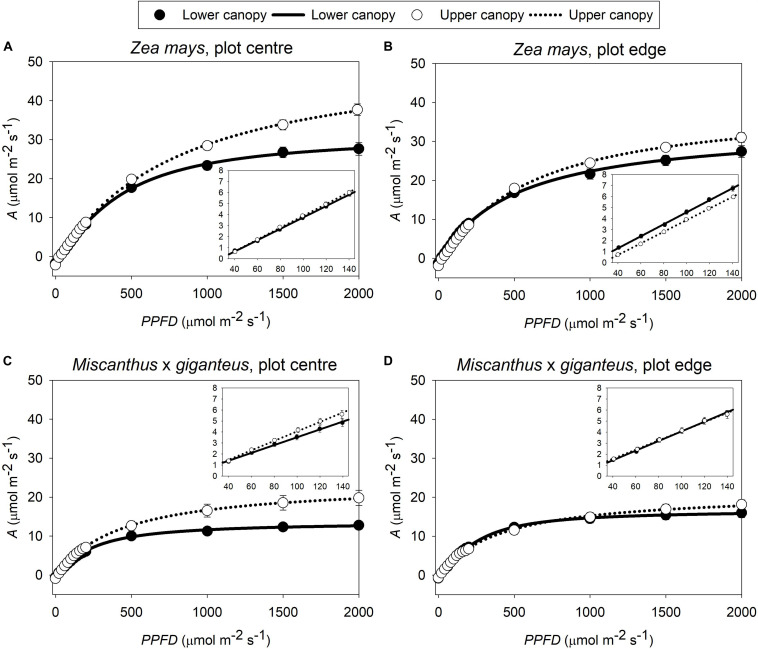
Average response curves of *A* to *PPFD* in both canopy positions (upper, lower) of **(A)** *Z. mays* in plot centre and **(B)** plot edge; and **(C)** *M.* x *giganteus* in plot centre and **(D)** plot edge (*n* = 8–16). Symbols give the mean *A* ± s.e. at each level of *PPFD*. Lines give the non-rectangular hyperbolae fit to these measurements. In each panel, the inset shows the light-limited section of the response curve (*PPFD* from 40 to 140 μmol m^–2^ s^–1^), used to estimate maximum quantum yields by linear regression: the slope of this regression gives the trait *ϕCO_2 max, app_*.

**FIGURE 2:**
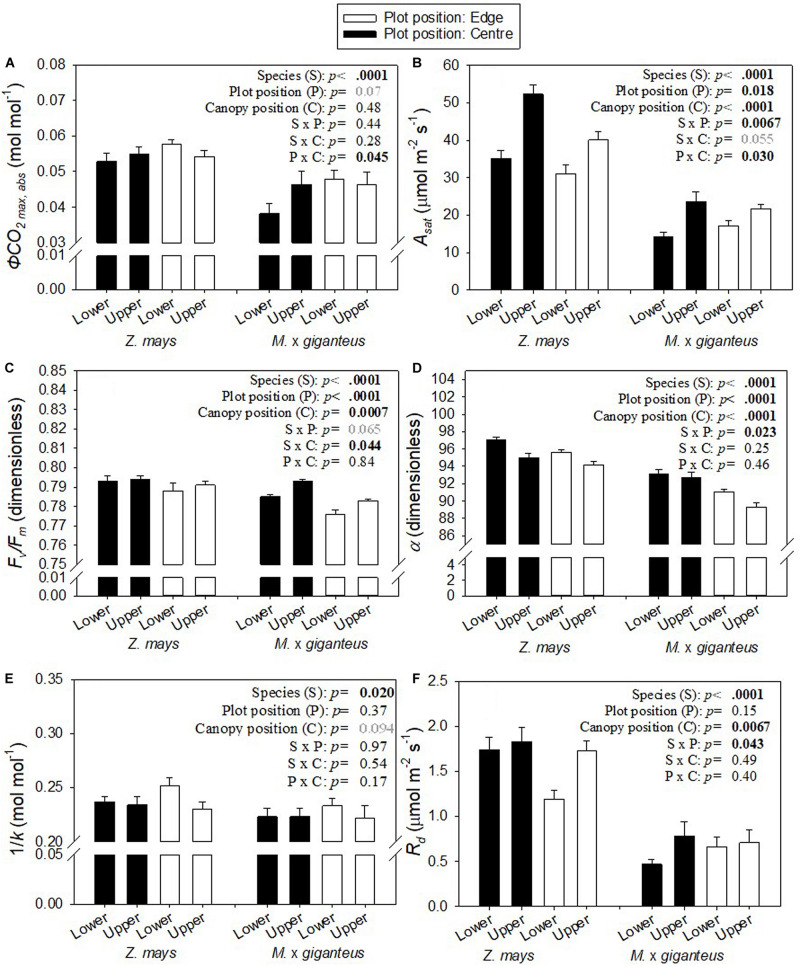
Mean ± s.e. for **(A)** *ϕCO_2 max,abs_*, **(B)** *A*_*sat*_, **(C)** *F_*v*_/F_*m*_*, **(D)** α, **(E)** 1/*k*, and **(F)** *R*_*d*_ for *Z. mays* and *M.* x *giganteus* for upper and lower canopy leaves in both plot positions (centre, edge) (*n* = 8–16). *p*-values are from ANOVA testing the fixed effects of species, plot position, canopy position, and all two-way interactions. Significant p-values (< 0.05) are in bold black. Marginally significant *p*-values (< 0.1) are in bold grey.

In this study, the three key measures of photosynthetic efficiency (*ϕCO_2 max, app_*, *ϕCO_2 max, abs_* and *ϕCO_2 max, abs PSII_*) all derive from the linear slope, at low *PPFD*, of the *A*-*PPFD* response curve (Figure 1). There was a significant interaction (*p* < 0.05) between canopy position and plot position for all three of these metrics (Figure 2A: P x C interaction, Supplementary Table S1: P x C interaction). This was because photosynthetic efficiency was greater at the top than the bottom of the canopy at the plot centre, while the opposite was seen at the plot edge where photosynthetic efficiency was slightly lower at the top than at the bottom of the canopy. Indeed, at the plot centre lower canopy leaves of both *Z. mays* and *M.* x *giganteus* showed a 2–18% reduction across all measures of photosynthetic efficiency compared to the upper canopy leaves (*ϕCO_2 max, app_*, *ϕCO_2 max, abs_*, *ϕCO_2 max, abs PSII_*, Figure 2A and Supplementary Table S1). In contrast, at the edge of the plots, the lower canopy leaves for both *Z. mays* and *M.* x *giganteus* showed 2–9% greater efficiency than the upper canopy leaves for the same measurements. In addition, *Z. mays* recorded significantly (*p* < 0.0001) and up to 43% greater values than *M.* x *giganteus* for *ϕCO_2 max, app_*, *ϕCO_2 max, abs_*, and *ϕCO_2 max, abs PSII_* (Figure 2A: S effect and Supplementary Table S1: S effect). Finally, *ϕCO_2 max, abs_* at the plot edge was marginally significantly (*p* = 0.07) and up to 25% greater than at the plot centre (Figure 2A: P effect).

The only other measure that showed a statistically significant interaction of plot and canopy position was *A*_*sat*_. *A*_*sat*_ was significantly (*p* < 0.0001) and up to 2-fold greater in *Z. mays* than *M.* x *giganteus* (Figure 2B, S effect), significantly (*p* = 0.018) greater at the centre than at the edge of the plot (Figure 2B, P effect), and significantly (*p* < 0.0001) greater at the top than at the bottom of the canopy (Figure 2B, C effect). The difference in *A*_*sat*_ between canopy levels was more pronounced at the centre than at the edge of plots, leading to a significant interaction (*p* = 0.03) of canopy position and plot position (Figure 2B: P x C interaction). Relative to the upper canopy, *A*_*sat*_ was decreased in the lower canopy by 30 and 40% in *Z. mays* and *M.* x *giganteus*, respectively, in the centre of the plots and by 23 and 21% in *Z. mays* and *M.* x *giganteus*, respectively, at the edge of the plots. *A*_*sat*_ showed significant interaction (*p* = 0.0067) of species and plot position (Figure 2B: S x P interaction), and a marginally significant interaction (*p* = 0.055) of species and canopy position (Figure 2B: S x C interaction). This was because differences in *A*_*sat*_ between canopy positions and between plot positions were more pronounced in *Z. mays* than in *M.* x *giganteus*.

There were statistically significant (*p* = 0.0007) decreases in *F*_*v*_/*F*_*m*_ in the lower canopy relative to the upper canopy, but in absolute terms this was a minor difference at less than 1% (Figure 2C: C effect). There were similarly small, but significant (*p* < 0.0001), decreases in *F*_*v*_/*F*_*m*_ at the edge relative to the centre (Figure 2C: P effect), and in *M.* x *giganteus* relative to *Z. mays* (Figure 2C: S effect). Differences in *F*_*v*_/*F*_*m*_ between canopy positions and between plot positions were slightly more pronounced in *M.* x *giganteus* than in *Z. mays*, resulting in a significant interaction (*p* = 0.044) of species and canopy position (Figure 2C: S x C interaction), and a marginally significant interaction (*p* = 0.065) of species and plot position (Figure 2C: S x P interaction).

Lower canopy leaves had significantly (*p* < 0.0001) and up to 2% greater *α* than upper canopy leaves (Figure 2D: C effect). *α* was also significantly (*p* < 0.0001) lower in *M.* x *giganteus* in comparison to *Z. mays* (Figure 2D: S effect) and significantly (*p* < 0.0001) greater at the plot centre than at the edge (Figure 2D: P effect). There was a significant interaction (*p* = 0.023) of species with plot position (Figure 2F: S x P interaction) because the difference in *α* between species was 5% at the edge of the plots and only 3% in the centre of the plots.

**FIGURE 3:**
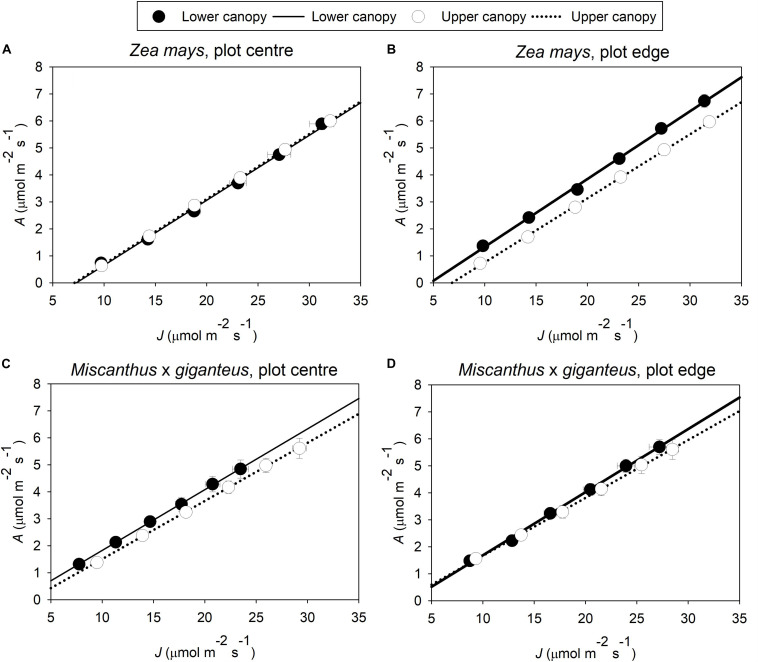
Average linear responses of *A* to *J* in **(A)** *Z. mays* in plot centre and **(B)** plot edge; and **(C)** *M.* x *giganteus* in plot centre and **(D)** plot edge (*n* = 8–16). Data is from light-limited measurements (*PPFD* from 40 to 140 μmol m^–2^ s^–1^). Symbols give the mean *A* ± s.e. and mean *J* ± s.e. at each level of *PPFD*. Lines give the best-fit linear regression; the slope of this regression gives the trait 1/*k*.

*1/k*, i.e., the ratio of *A* to *J* (Figure 3), was marginally significantly (*p* = 0.094), and up to 9% greater in lower canopy leaves than upper canopy leaves (Figure 2E: C effect). *1/k* was also significantly (*p* = 0.02) and 4–8% greater in *Z. mays* than in *M.* x *giganteus* (Figure 2E: S effect).

*R*_*d*_ was significantly (*p* = 0.0067) and 5–66% greater in upper canopy leaves than lower canopy leaves (Figure 2F: C effect). In line with the higher *A*_*sat*_, *R*_*d*_ was also significantly (*p* < 0.0001) greater in *Z. mays* than in *M.* x *giganteus* (Figure 2F: S effect). The difference in *R*_*d*_ between species was less pronounced at the edge than at the centre of the plots, resulting in a significant (*p* = 0.043) interaction between species and plot position (Figure 2F: S x P interaction). There was 144 and 80% difference between species for upper and lower canopy leaves at the plot edge, respectively, compared to 135 and 270% difference between species for upper and lower canopy leaves at the plot centre.

## Discussion

### Reduced Maximum Quantum Yield of CO_2_ Assimilation Is Not Caused by Increased Leaf Age

In a self-shading crop canopy, the optimal response to shade would be to maintain or increase *ϕCO_2 max, abs_* and increase α in order to maximize photosynthesis in light limited conditions. This would increase the linear slope of the response of *A* to *PPFD* at low light. This response is observed in shade adapted C_3_ plants and in C_3_ cereal crops (Givnish, 1988; Beyschlag et al., 1990; Hoyaux et al., 2008). However, the two C_4_ crops *Z. mays* and *M.* x *giganteus* studied here, show decreased *ϕCO_2 max, abs_* in the lower canopy at the plot centre, but not at the plot edge. This suggests that loss of *ϕCO_2 max, abs_* in shade leaves was not due to leaf age, since leaf age was equivalent across plot positions for each species and canopy position. Understanding the basis for this maladaptive response in photosynthetic efficiency is important, as it costs an estimated 10% of potential canopy CO_2_ assimilation in the field (Pignon et al., 2017).

If not age, then some environmental factor must trigger the decline in *ϕCO_2 max, abs_* of these shaded leaves. The most obvious environmental change between the top and bottom of the canopy is the light environment, with lower leaves receiving less light and an altered spectral distribution, depleted of red and blue and enriched in far-red wavelengths (Sattin et al., 1994). The hypothesis that self-shading is the primary cause for the loss of *ϕCO_2 max, abs_* in shade leaves of these C_4_ NADP-ME crops is supported by the following observations: (1) when comparing both studied species, the loss of *ϕCO_2 max, abs_* in shade leaves at the plot centre was more pronounced in *M.* x *giganteus*, which produces a denser canopy with considerably more self-shading than *Z. mays*. Profiles of canopy light interception in field stands of both species show that the lowest photosynthetically active leaves of *Z. mays* receive as much as twice the incident *PPFD* compared to equivalent leaves in *M.* x *giganteus* (Pignon et al., 2017). (2) In a previous study comparing two field-grown sugarcane varieties with high and low self-shading, photosynthetic light response curves measured at the top and bottom of the canopy produced contrasting results in the response of *A* to *PPFD* at low *PPFD* (<500 μmol m^–2^ s^–1^) (Marchiori et al., 2014). In the low self-shading variety, *A* at low *PPFD* was slightly greater at the bottom than at the top of the canopy, while the opposite was seen in the high self-shading variety. These studies implemented shade acclimation under realistic field conditions, which produce different results than artificial shading including altered spectral light composition and increased incidence of sun and shade flecks (Pearcy, 1990; Bellasio and Griffiths, 2014; Yabiku and Ueno, 2019).

These findings are important in light of recent efforts to develop crops with a more even vertical light distribution, where either more erect (Perez et al., 2018; San et al., 2018) or more transparent (Slattery et al., 2016; Walker et al., 2018) leaves allow more light to filter to the bottom of the canopy, ultimately increasing canopy photosynthesis (Zhu et al., 2010). The benefits of this type of canopy manipulation could be 2-fold in NADP-ME C_4_ crops such as *Z. mays*, *M.* x *giganteus*, sugarcane or sorghum, providing both increased light to drive more photosynthesis and minimizing the loss of photosynthetic efficiency in lower canopy leaves.

Apart from light, temperature is one other important change in microclimate between canopy and plot positions, as shaded leaves can be expected to be cooler. However, temperature is a less likely candidate than light to explain the lost photosynthetic efficiency of shaded leaves seen at the plot centre in the present study. In C_3_ plants, *ϕCO_2 max, abs_* is highly temperature-sensitive, primarily due to increased photorespiration at high temperatures (Ehleringer and Bjorkman, 1977; Long and Spence, 2013). In contrast, due to the C_4_ cycle’s suppression of photorespiration, *ϕCO_2 max, abs_* has been found to be constant with temperature from 15 to 40°C in C_4_ species such as *Atriplex rosea* (Ehleringer and Bjorkman, 1977) and *Alloteropsis semialata* (Osborne et al., 2008). Although loss of *ϕCO_2 max, abs_* has been observed in NADP-ME C_4_ grasses such as *Z. mays* due to photodamage during long-term exposure to a combination of high light and cool temperatures (<15°C) (Long and Spence, 2013), this is unlikely to have occurred in the warm summer months during which the present study took place, with maximum daily air temperatures ranging from 19.5 to 33°C at the time measurements were taken. Indeed, since the lower canopy leaves on the exposed southern edge of the stands were exposed to higher light intensities than the shaded lower leaves in the centre of the stands, the expectation would be of a lower *ϕCO_2 max, abs_* due to photodamage in the exposed lower canopy leaves, yet the opposite was found.

### Physiological Traits Underpinning Maximum Quantum Yield of CO_2_ Assimilation

Under limiting light, reduced α in lower canopy leaves would limit the amount of incident light made available for use within the leaf, and would result in reduced maximum quantum yield on an incident light basis (i.e., *ϕCO_2 max, app_*). The fact that α increased in lower canopy leaves shows that in fact their light absorption was improved, not impaired. This pattern in α, along with *R*_*d*_ and *A*_*sat*_, matches established mechanisms of acclimation to low light, as shade leaves: (1) reduce *R*_*d*_, (2) remobilize N away from photosynthetic enzymes and toward chlorophyll to improve *α* under limiting light, and (3) translocate N to the upper canopy so sun leaves can increase photosynthetic enzyme content and improve *A*_*sat*_ (Boardman, 1977; Chen et al., 2014; Niinemets, 2016b; D’Odorico et al., 2018).

Because of the difference in light availability between sun and shade leaves, shade leaves benefit from partitioning relatively more N toward chlorophyll, compared to sun leaves that partition much more N toward photosynthetic enzymes. Therefore, while shade leaves typically reallocate the N stored in their photosynthetic enzymes and decrease total N content, this primarily results in a loss of *A*_*sat*_, while the apparent maximum quantum yield (*ϕCO_2 max, app_*) rises due to increased chlorophyll and, in turn, increased α. The unusual feature in this study is that *ϕCO_2 max, app_* falls despite an increase in α – hence our use of the term maladaptive to describe the response of studied shade leaves to low light. Also, as *ϕCO_2 max, abs_* is measured on an absorbed light basis and derived from the initial linear slope of the light response curve, it is by definition where *A* is strictly light-limited, ruling out any limitation by N or protein amounts which primarily affect *A*_*sat*_ (Hikosaka and Terashima, 1995). In fact, the maximum quantum yield of CO_2_ assimilation corrected for chlorophyll content was equivalent in N-stressed and control maize plants (Lu and Zhang, 2000).

Efficient energy transfer at PSII is essential to power photosynthesis under limiting light. *F*_*v*_/*F*_*m*_ is an effective probe to determine whether damage to PSII has occurred (Baker, 2008). However, the <1% loss of *F*_*v*_/*F*_*m*_ observed here in lower canopy leaves cannot explain the much more substantial losses in *ϕCO_2 max,abs_*.

1/*k*, i.e., the ratio of *A* to the rate of linear electron transport through PSII (*J*) at low light, is decreased when the energetic compounds NADPH, reduced ferredoxin, and ATP, produced through linear electron flux, are diverted away from photosynthetic carbon metabolism and into other energy-consuming processes (e.g., nitrogen metabolism, Mehler reaction) (Delatorre et al., 1991; Baker, 2008). This is observed as a reduced slope of the linear relationship of *A* to *J*. Under limiting light, this will cause a decline in *ϕCO_2 max, abs_*. However, in lower canopy leaves, 1/*k* was greater than at the top of the canopy, implying leaves at the bottom of the canopy actually had fewer, not more, alternative energy sinks to photosynthetic carbon metabolism. In fact, alternative energy sinks overall were minimal: 1/*k* was always close to the theoretical maximum of 0.25 mol mol^–1^, i.e., for each mol CO_2_ assimilated, a theoretical minimum of *k* = 4 mol electron equivalents must be produced through linear electron flux when there are no alternative energy sinks (Baker, 2008).

One possible explanation for loss of *ϕCO_2 max, abs_* without reduced 1/*k* is that lower canopy leaves in the plot centre did have increased alternative sinks, but these were not detected by the leaf fluorescence measurements. One caveat of PSII fluorescence is that the signal is primarily obtained from PSII closest to the leaf surface, with less contribution from PSII deeper in the leaf. Therefore 1/*k* is obtained from *A* throughout the entire leaf cross-section, and *J* obtained from PSII fluorescence at the leaf surface. If alternative energy sinks diverted NADPH and ATP from deeper PSII, this could result in a decrease of *ϕCO_2 max, abs_* without an apparent effect to 1/*k*. Additionally, 1/*k* only measures the partitioning toward *A* of NADPH and ATP produced through linear electron flux. ATP can also be produced through cyclic electron flux around PSI, a process which bypasses PSII and produces only ATP (von Caemmerer, 2000). Alternative energy sinks for the ATP produced through cyclic electron flux would not be reflected in 1/*k*, since 1/*k* is based on the photochemical efficiency of PSII and not PSI. For instance, shaded leaves of field-grown *M.* x *giganteus* show signs of increased leakage of CO_2_ from bundle-sheath cells, which should incur additional ATP consumption to power C_4_ overcycling of CO_2_ (Kromdijk et al., 2008). However, C_4_ NADP-ME grasses including *Z. mays* showed increased photon partitioning to PSI, but no significant change in cyclic electron flux, when grown in the shade (Ver Sagun et al., 2019).

*ϕCO_2 max, abs_* measured in non-stressed conditions is typically well conserved across various species (Long et al., 1993). Surprisingly, here *Z. mays* showed *ϕCO_2 max, abs_* 23% greater than *M.* x *giganteus*. This may be explained in part by the greater *F*_*v*_/*F*_*m*_ and 1/*k* in *Z. mays* relative to *M.* x *giganteus*. In previous measurements on nearby plots of the same species, *ϕCO_2 max, abs_* of *M.* x *giganteus* and *Z. mays* were within just 9% of one another (Pignon et al., 2017), suggesting the greater inter-species difference observed here may be an effect of different location or growing season.

### Potential Effects of Breeding and Management on Maximum Quantum Yield of CO_2_ Assimilation

These results raise the question of why such productive crops show a maladaptive acclimation to shade. *Zea mays* in particular is being grown at ever greater densities (Lobell et al., 2014), resulting in increased leaf area indices and self-shading, but these high densities are a recent construct of cultivation. The ancestors of cultivated *Z. mays* grew largely as isolated plants in semi-arid and nutrient limited environments, such that they would have evolved as plants in which most or all leaves were exposed to full sunlight and shading was rare. Similarly, *Miscanthus* spp. often occur as single tall clumps, standing above surrounding plants and so too would experience relatively little shading, compared to field production stands. Having evolved as sun plants, there may have been insufficient time for them to adapt to the recent production in dense stands.

Although both species are part of the same C_4_ evolutionary clade, modern *Z. mays* hybrids have been subject to centuries of selection for productivity, which has been particularly intense in the last 50 years, while *M. x giganteus* is only just emerging as a crop. This may suggest that there is variability that could be selected to overcome this significant Achilles heel in this important group of crops. *Z. mays* is considered to have diverged in the evolution of the Andropogoneae before divergence of the genera *Saccharum, Sorghum* and *Miscanthus* (Kim et al., 2014; Singh et al., 2019). The occurrence of this maladaptation in both *Z. mays* and *M. x giganteus* suggests that the major crops sorghum and sugarcane are likely similarly affected. Given that *Z. mays* accounts for more cereal grain than any other crop globally, overcoming this maladaptation to shade would contribute very significantly toward meeting the 60% increase in food demand anticipated for mid-century (Long et al., 2015; FAO, 2017).

## Data Availability Statement

All original data is freely available without restrictions from the Illinois Data Bank, 10.13012/B2IDB-4821336_V1.

## Author Contributions

RC and ER collected the physiological data and wrote the manuscript. CP supervised the experiment, performed the statistical analysis, and assisted in manuscript writing. SL conceived the experiment and assisted in manuscript writing.

## Conflict of Interest

The authors declare that the research was conducted in the absence of any commercial or financial relationships that could be construed as a potential conflict of interest.
