# Supplementary material for: Light, Not Age, Underlies the Maladaptation of Maize and Miscanthus Photosynthesis to Self-Shading
Source: Front Plant Sci. 2020 Jun 24;11:783. doi: 10.3389/fpls.2020.00783 (PMC7358635; doi:10.3389/fpls.2020.00783)
Supplement: Supplementary file 1 [file Table_1.DOCX]

# Supplementary Material

Supplementary Table S1: Mean ± s.e. for *ΦCO_2 max, app_* and *ΦCO_2 max, abs PSII_*, for *Z. mays* and *M.* x *giganteus* in both plot positions (centre, edge) and for both canopy positions (upper, lower). *p*-values are shown from ANOVA testing the fixed effects of species, plot position, canopy position, and all two-way interactions.

| Species | Plot position | Canopy position | *Φ_CO2 max, app_* | *Φ_CO2 max, abs PSII_* |
| --- | --- | --- | --- | --- |
| *Z. mays* | Centre | Upper | 0.0533 ± 0.0014 | 0.063 ± 0.0022 |
|  |  | Lower | 0.0506 ± 0.0018 | 0.0619 ± 0.0023 |
|  |  |  |  |  |
|  | Edge | Upper | 0.0525 ± 0.0014 | 0.0611 ± 0.0019 |
|  |  | Lower | 0.0538 ± 0.0013 | 0.0674 ± 0.0018 |
|  |  |  |  |  |
| *M.* x *giganteus* | Centre | Upper | 0.0429 ± 0.0033 | 0.0572 ± 0.0036 |
|  |  | Lower | 0.0355 ± 0.0025 | 0.049 ± 0.0032 |
|  |  |  |  |  |
|  | Edge | Upper | 0.0414 ± 0.0031 | 0.0568 ± 0.0035 |
|  |  | Lower | 0.0437 ± 0.0023 | 0.0579 ± 0.0028 |

| ANOVA fixed effect | *Φ_CO2 max, app_* | *Φ_CO2 max, abs PSII_* |
| --- | --- | --- |
| Species (S) | <.0001 | 0.0001 |
| Plot position (P) | 0.14 | 0.14 |
| Canopy position (C) | 0.27 | 0.81 |
| S x P | 0.49 | 0.54 |
| S x C | 0.50 | 0.12 |
| P x C | 0.035 | 0.038 |
